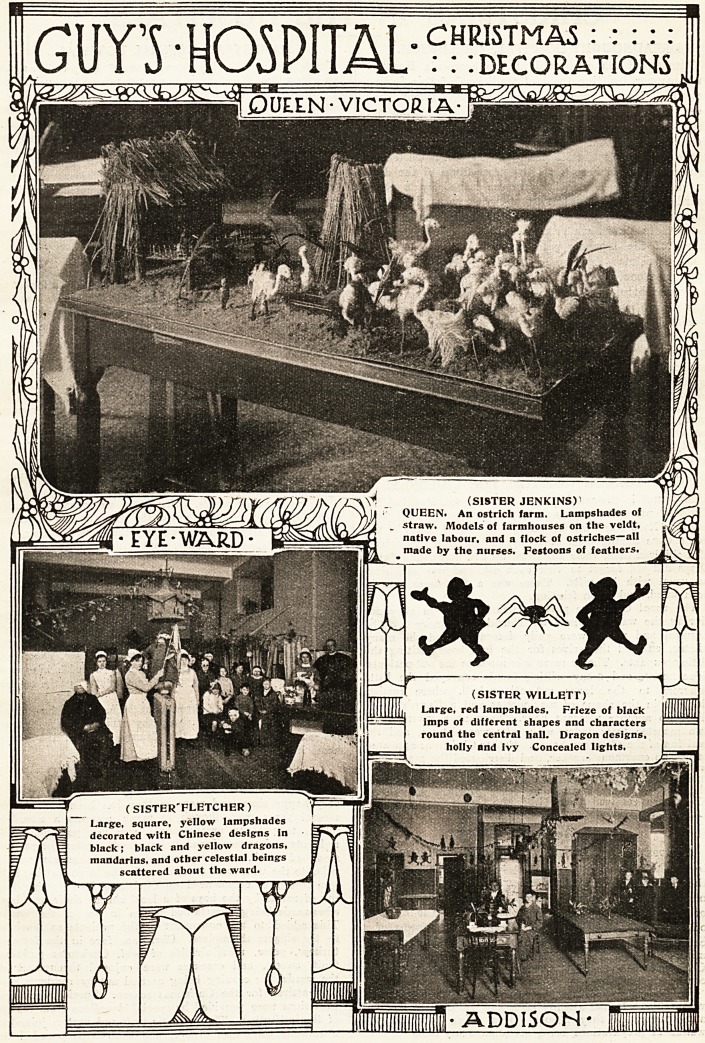# Guy's Hospital: Christmas Decorations

**Published:** 1920-01-10

**Authors:** 


					January iIO, 1920. THE HOSPITAL 3,45
GUYS ? HOSPITAL :DECOROTONj
MQ THE HOSPITAL January 10, 1920.
January 10, 1920. THE HOSPITAL 347

				

## Figures and Tables

**Figure f1:**
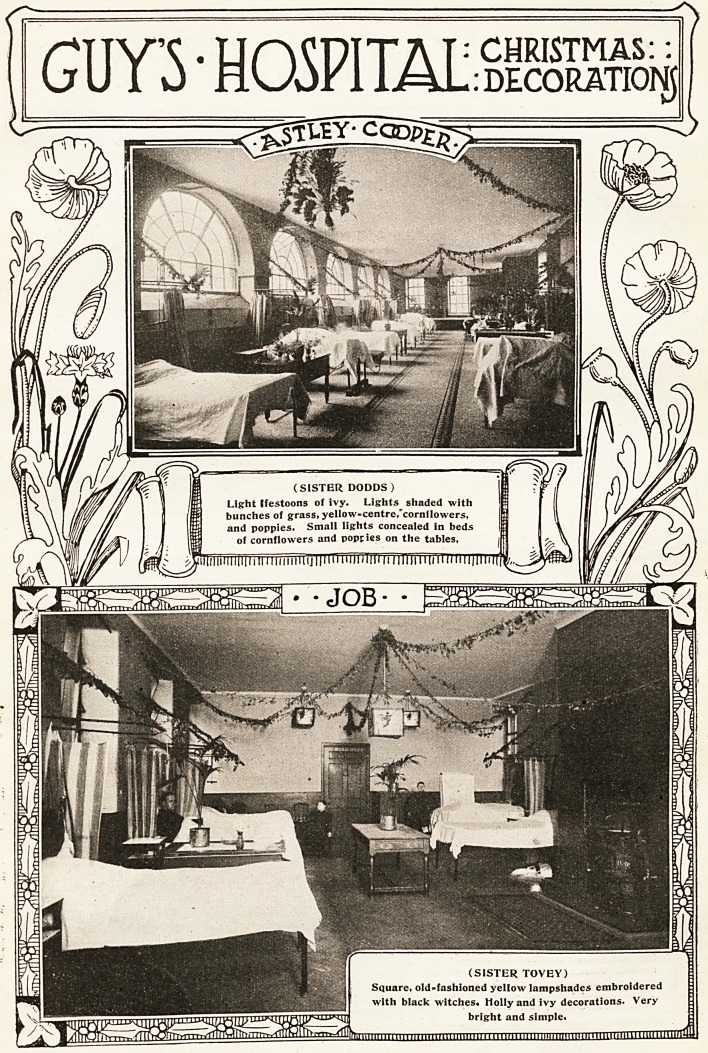


**Figure f2:**
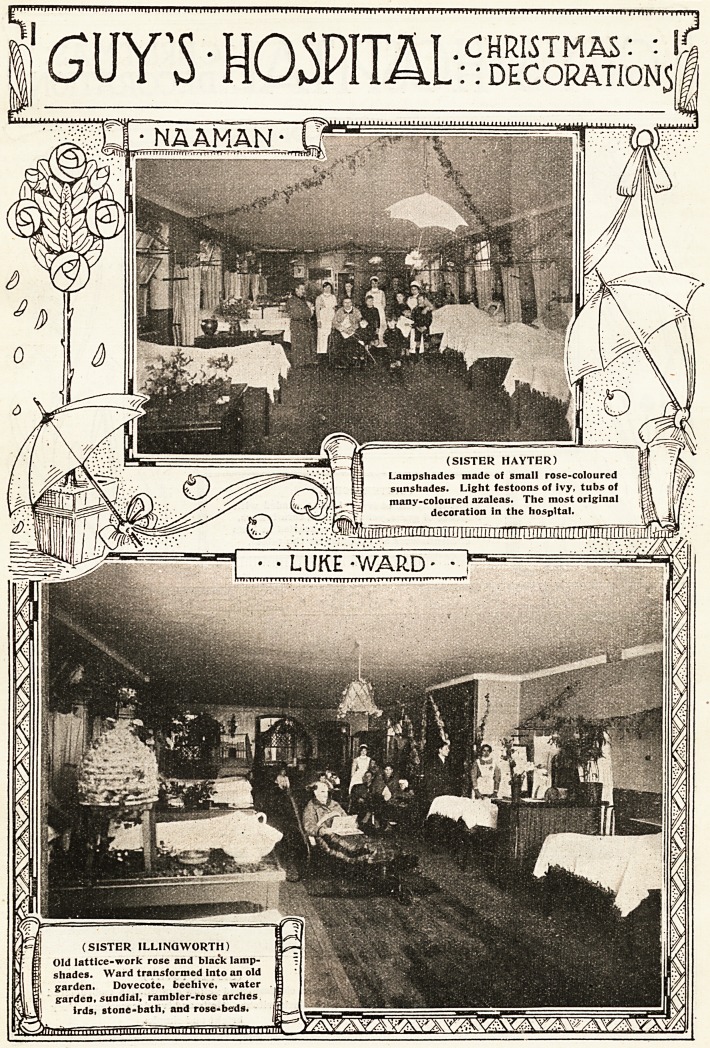


**Figure f3:**